# The genome sequence of a hoverfly, *Cheilosia grossa *(Fallén, 1817)

**DOI:** 10.12688/wellcomeopenres.23189.1

**Published:** 2024-10-21

**Authors:** Ryan Mitchell, Steven Falk, Katie J. Woodcock, Natural History Museum Genome Acquisition Lab, University of Oxford and Wytham Woods Genome Acquisition Lab, Darwin Tree of Life Barcoding collective, Wellcome Sanger Institute Tree of Life Management, Samples and Laboratory team, Wellcome Sanger Institute Scientific Operations: Sequencing Operations, Wellcome Sanger Institute Tree of Life Core Informatics team, Tree of Life Core Informatics collective, Darwin Tree of Life Consortium

**Affiliations:** 1 Independent researcher, Sligo, County Sligo, Ireland; 2 Independent researcher, Kenilworth, England, UK; 3 Tree of Life, Wellcome Sanger Institute, Hinxton, England, UK

**Keywords:** Cheilosia grossa, hoverfly, genome sequence, chromosomal, Diptera

## Abstract

We present a genome assembly from an individual male *Cheilosia grossa* (a hoverfly; Arthropoda; Insecta; Diptera; Syrphidae). The genome sequence has a total length of 362.40 megabases. Most of the assembly is scaffolded into 6 chromosomal pseudomolecules, including the X sex chromosome. The mitochondrial genome has also been assembled and is 17.81 kilobases in length. Gene annotation of this assembly on Ensembl identified 20,196 protein-coding genes.

## Species taxonomy

Eukaryota; Opisthokonta; Metazoa; Eumetazoa; Bilateria; Protostomia; Ecdysozoa; Panarthropoda; Arthropoda; Mandibulata; Pancrustacea; Hexapoda; Insecta; Dicondylia; Pterygota; Neoptera; Endopterygota; Diptera; Brachycera; Muscomorpha; Eremoneura; Cyclorrhapha; Aschiza; Syrphoidea; Syrphidae; Eristalinae; Rhingiini; *Cheilosia*; *Cheilosia grossa* (Fallén, 1817) (NCBI:txid273442).

## Background

*Cheilosia grossa* (Fallén, 1817) is a large, fluffy hoverfly species that is frequently observed across the UK and Ireland, as well as Northern and Central Europe ( Ball & Morris, 2015; van Veen, 2014). This early spring species can be found between March and May, peaking in numbers during late March and early April ( Stubbs & Falk, 2002). The characteristic presence of orange tibiae and black antennae combined with their furry appearance make confusion with other species unlikely ( Ball & Morris, 2015).

As adults *C. grossa* can be observed in a range of habitats including woodland rides and edges, heathlands and damp meadows, particularly in areas where thistles are abundant ( Stubbs & Falk, 2002). Adults can be spotted visiting willows such as *Salix caprea* (goat willow) and other early spring flowering plants, including blackthorn, colt’s-foot and dandelion ( Ball & Morris, 2015). Males have been documented hovering at head height over open ground and can, on occasion, form loose swarms at around 2 to 6 metres above the ground ( Ball & Morris, 2000; Stubbs & Falk, 2002). During early spring female *C. grossa* have been recorded laying eggs upon early flowering thistle spikes, with a noted preference for marsh and spear thistle ( Ball & Morris, 2000). Within a few days the phytophagous larvae emerge and begin tunnelling within the roots and stems, larval infestation is indicated by the sprouting of multiple stems and stunted plant growth ( Ball & Morris, 2000; Rotheray, 1993). Larvae tunnel out of the thistle root during late August and overwinter as pupae in soil, before emerging as adults the following spring for the life cycle to begin again ( Rotheray, 1993).

Records for adult *C. grossa* appear relatively sparse, likely due to a combination of their early spring activity and their tendency to fly high making them difficult to observe ( Ball & Morris, 2015). However, larval records suggest that this species is more common and widespread than adult sightings alone would indicate ( Ball & Morris, 2015).

The completed genome sequence for *Cheilosia grossa* presented here provides a valuable tool to further the study of this often overlooked hoverfly species.

## Genome sequence report

**Figure 1.**
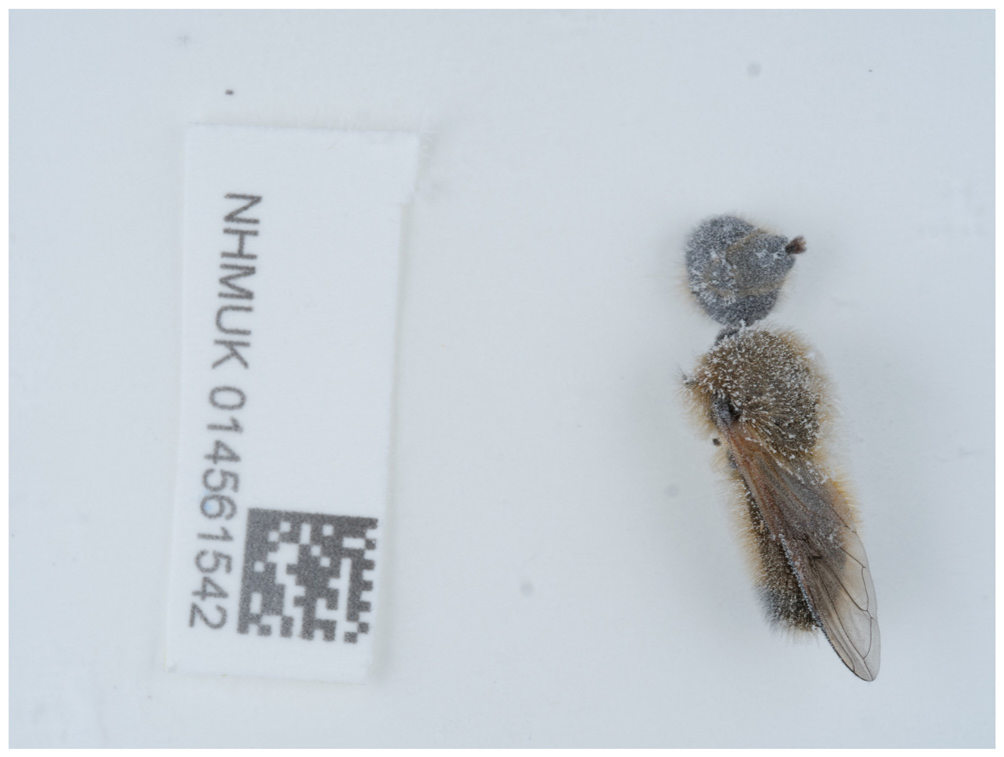
Photograph of the *Cheilosia grossa* (idCheGros1) specimen used for genome sequencing.

The genome of an adult male specimen of *Cheilosia grossa* ( Figure 1) was sequenced using Pacific Biosciences single-molecule HiFi long reads, generating a total of 29.36 Gb (gigabases) from 2.85 million reads, providing approximately 83-fold coverage. Primary assembly contigs were scaffolded with chromosome conformation Hi-C data, which produced 128.65 Gb from 852.00 million reads, yielding an approximate coverage of 355-fold. Specimen and sequencing information is summarised in Table 1.

**Table 1.** Specimen and sequencing data for *Cheilosia grossa*.

Project information			
**Study title**	*Cheilosia grossa*		
**Umbrella BioProject**	PRJEB63511		
**BioSample**	SAMEA110054238		
**NCBI taxonomy ID**	273442		
Specimen information			
**Technology**	**ToLID**	**BioSample accession**	**Organism part**
**PacBio long read sequencing**	idCheGros1	SAMEA14448159	abdomen
**Hi-C sequencing**	idCheGros1	SAMEA14448161	head and thorax
**RNA sequencing**	idCheGros2	SAMEA113427013	abdomen
Sequencing information			
**Platform**	**Run accession**	**Read count**	**Base count (Gb)**
**Hi-C Illumina NovaSeq 6000**	ERR11606342	8.52e+08	128.65
**PacBio Sequel IIe**	ERR11641066	2.85e+06	29.36
**RNA Illumina NovaSeq 6000**	ERR12245578	7.65e+07	11.56

**Figure 2.**
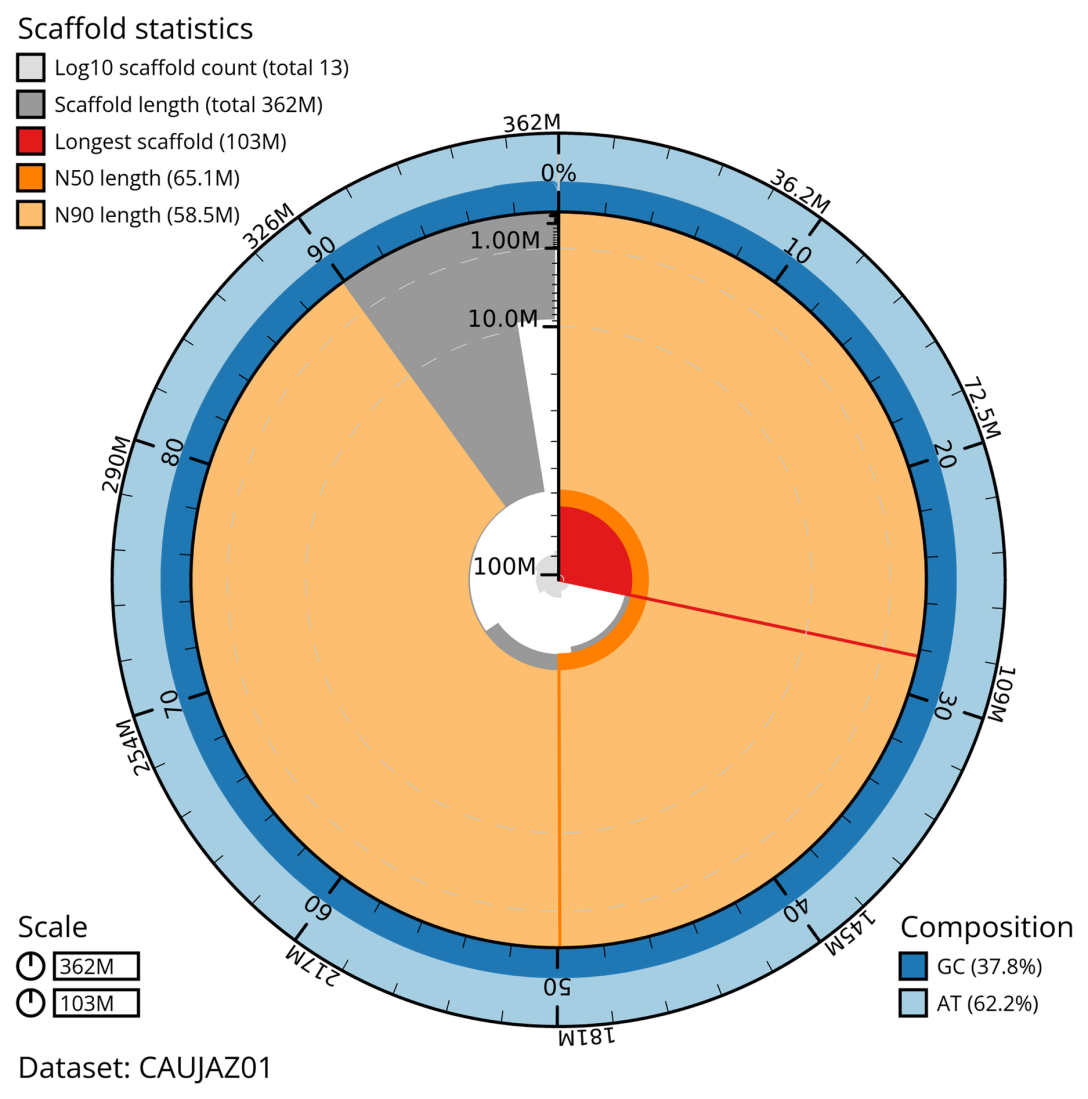
Genome assembly of *Cheilosia grossa*, idCheGros1.1: metrics. The BlobToolKit snail plot shows N50 metrics and BUSCO gene completeness. The main plot is divided into 1,000 size-ordered bins around the circumference with each bin representing 0.1% of the 362,407,107 bp assembly. The distribution of scaffold lengths is shown in dark grey with the plot radius scaled to the longest scaffold present in the assembly (102,793,891 bp, shown in red). Orange and pale-orange arcs show the N50 and N90 scaffold lengths (65,050,764 and 58,539,889 bp), respectively. The pale grey spiral shows the cumulative scaffold count on a log scale with white scale lines showing successive orders of magnitude. The blue and pale-blue area around the outside of the plot shows the distribution of GC, AT and N percentages in the same bins as the inner plot. A summary of complete, fragmented, duplicated and missing BUSCO genes in the diptera_odb10 set is shown in the top right. An interactive version of this figure is available at https://blobtoolkit.genomehubs.org/view/CAUJAZ01/dataset/CAUJAZ01/snail.

**Figure 3.**
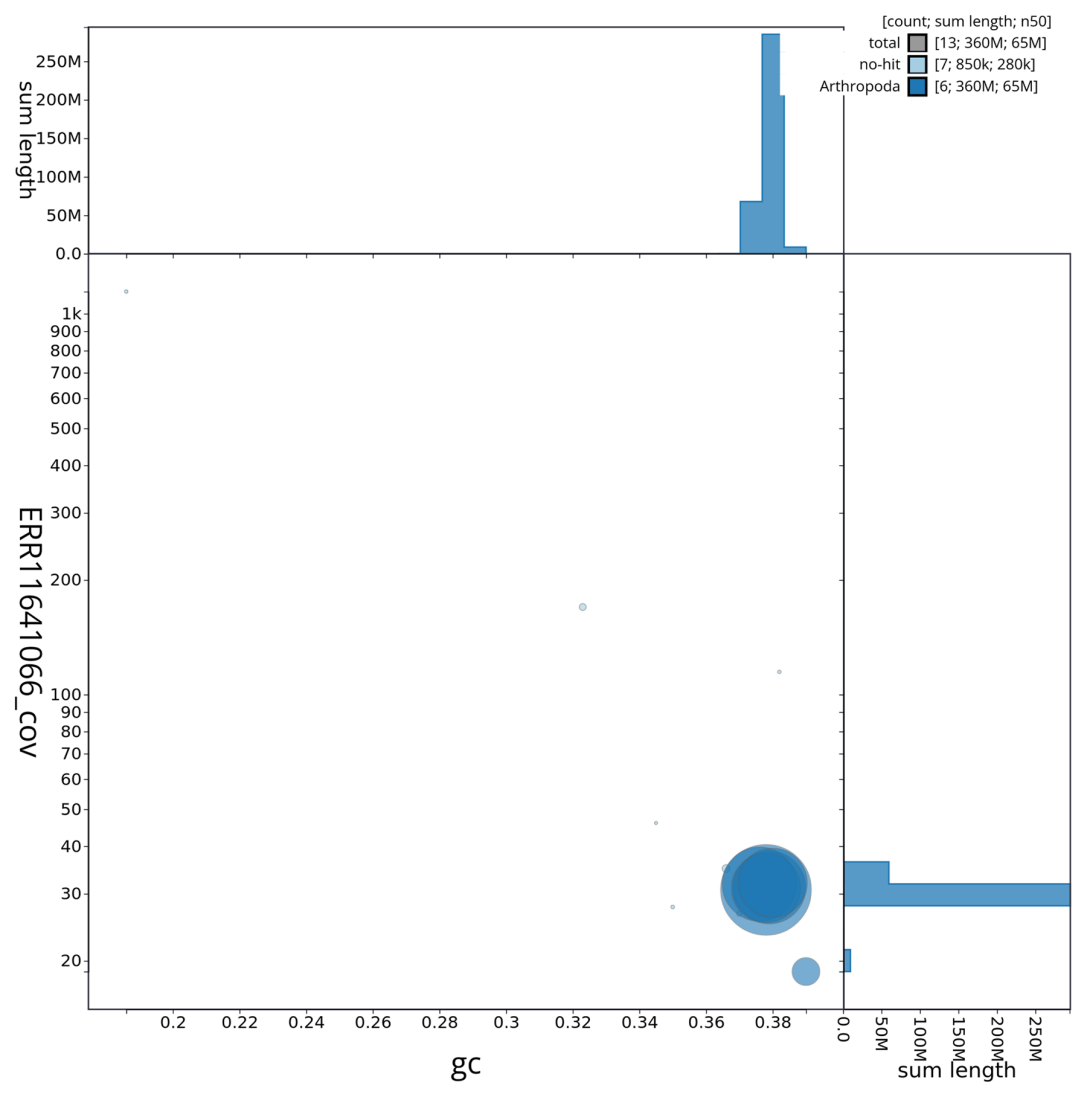
Blob plot of base coverage against GC proportion for sequences in the assembly idCheGros1.1. Sequences are coloured by phylum. Circles are sized in proportion to sequence length. Histograms show the distribution of sequence length sum along each axis. An interactive version of this figure is available at https://blobtoolkit.genomehubs.org/view/CAUJAZ01/dataset/CAUJAZ01/blob.

**Figure 4.**
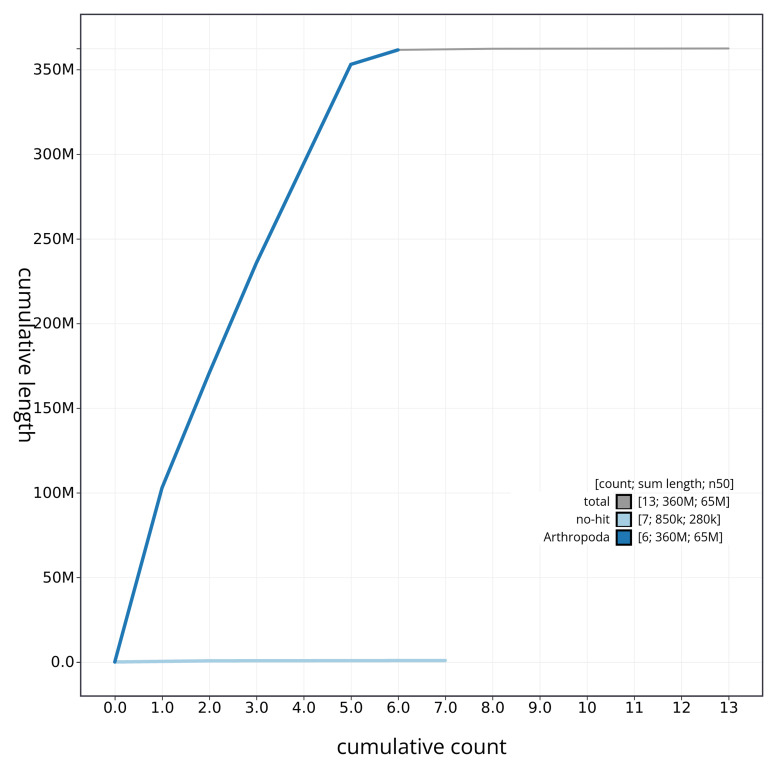
Genome assembly of *Cheilosia grossa* idCheGros1.1: BlobToolKit cumulative sequence plot. The grey line shows cumulative length for all sequences. Coloured lines show cumulative lengths of sequences assigned to each phylum using the buscogenes taxrule. An interactive version of this figure is available at https://blobtoolkit.genomehubs.org/view/CAUJAZ01/dataset/CAUJAZ01/cumulative.

**Figure 5.**
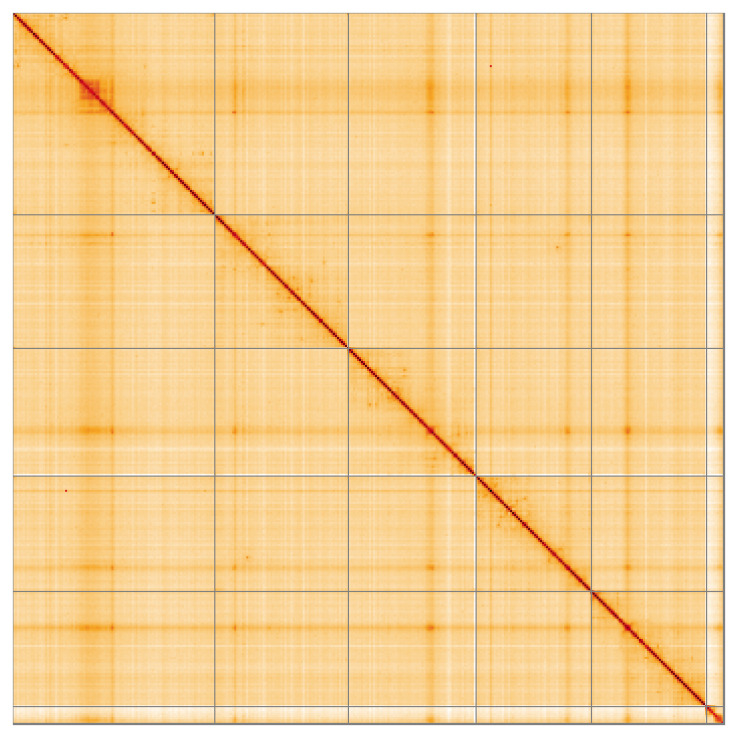
Genome assembly of *Cheilosia grossa* idCheGros1.1: Hi-C contact map of the idCheGros1.1 assembly, visualised using HiGlass. Chromosomes are shown in order of size from left to right and top to bottom. An interactive version of this figure may be viewed at https://genome-note-higlass.tol.sanger.ac.uk/l/?d=BwZWlNXsRS-pV40eYunn9g.

Manual assembly curation corrected 14 missing joins or mis-joins, reducing the scaffold number by 35.0%. The final assembly has a total length of 362.40 Mb in 12 sequence scaffolds with a scaffold N50 of 65.1 Mb ( Table 2). The total count of gaps in the scaffolds is 96. The snail plot in Figure 2 provides a summary of the assembly statistics, while the distribution of assembly scaffolds on GC proportion and coverage is shown in Figure 3. The cumulative assembly plot in Figure 4 shows curves for subsets of scaffolds assigned to different phyla. Most (99.76%) of the assembly sequence was assigned to 6 chromosomal-level scaffolds, representing 5 autosomes and the X sex chromosome. Chromosome-scale scaffolds confirmed by the Hi-C data are named in order of size ( Figure 5; Table 3). The X chromosome was identified based on half coverage. Y chromosome expected but could not be identified in the assembly. While not fully phased, the assembly deposited is of one haplotype. Contigs corresponding to the second haplotype have also been deposited. The mitochondrial genome was also assembled and can be found as a contig within the multifasta file of the genome submission.

**Table 2.** Genome assembly data for *Cheilosia grossa*, idCheGros1.1.

Genome assembly		
Assembly name	idCheGros1.1	
Assembly accession	GCA_963082955.1	
*Accession of alternate haplotype*	*GCA_963082755.1*	
Span (Mb)	362.40	
Number of contigs	109	
Contig N50 length (Mb)	5.4	
Number of scaffolds	12	
Scaffold N50 length (Mb)	65.1	
Longest scaffold (Mb)	102.79	
Assembly metrics *		*Benchmark*
Consensus quality (QV)	71.6	*≥ 50*
*k*-mer completeness	100.0%	*≥ 95%*
BUSCO **	C:96.9%[S:96.6%,D:0.3%], F:0.9%,M:2.2%,n:3,285	*C ≥ 95%*
Percentage of assembly mapped to chromosomes	99.76%	*≥ 95%*
Sex chromosomes	X	*localised homologous pairs*
Organelles	Mitochondrial genome: 17.81 kb	*complete single alleles*
Genome annotation of assembly GCA_963082955.1 at Ensembl		
Number of protein-coding genes	20,196	
Number of gene transcripts	20,893	

* Assembly metric benchmarks are adapted from column VGP-2020 of “Table 1: Proposed standards and metrics for defining genome assembly quality” from Rhie *et al.* (2021). ** BUSCO scores based on the diptera_odb10 BUSCO set using version 5.3.2. C = complete [S = single copy, D = duplicated], F = fragmented, M = missing, n = number of orthologues in comparison. A full set of BUSCO scores is available at https://blobtoolkit.genomehubs.org/view/CAUJAZ01/dataset/CAUJAZ01/busco.

**Table 3.** Chromosomal pseudomolecules in the genome assembly of *Cheilosia grossa*, idCheGros1.

INSDC accession	Name	Length (Mb)	GC%
OY720144.1	1	102.79	38.0
OY720145.1	2	67.88	37.5
OY720146.1	3	65.05	38.0
OY720147.1	4	58.71	38.0
OY720148.1	5	58.54	38.0
OY720149.1	X	8.58	39.0
OY720150.1	MT	0.02	18.5

The estimated Quality Value (QV) of the final assembly is 71.6 with *k*-mer completeness of 100.0%, and the assembly has a BUSCO v5.3.2 completeness of 96.9% (single = 96.6%, duplicated = 0.3%), using the diptera_odb10 reference set ( *n* = 3,285).

Metadata for specimens, BOLD barcode results, spectra estimates, sequencing runs, contaminants and pre-curation assembly statistics are given at https://links.tol.sanger.ac.uk/species/273442.

## Genome annotation report

The *Cheilosia grossa* genome assembly (GCA_963082955.1) was annotated at the European Bioinformatics Institute (EBI) on Ensembl Rapid Release. The resulting annotation includes 20,893 transcribed mRNAs from 20,196 protein-coding genes ( Table 2; https://rapid.ensembl.org/Cheilosia_grossa_GCA_963082955.1/Info/Index). The average transcript length is 4,020.37. There are 1.03 coding transcripts per gene and 3.70 exons per transcript.

## Methods

### Sample acquisition and DNA barcoding

An adult male *Cheilosia grossa* (specimen ID NHMUK014561542, ToLID idCheGros1) was collected from Besselsleigh Wood, England, UK (latitude 51.72, longitude –1.30) on 2021-03-22. The specimen was collected and identified by Ryan Mitchell (independent researcher) and preserved by dry freezing at –80°C.

The specimen used for RNA sequencing (specimen ID Ox003365, ToLID idCheGros2) was an adult specimen collected from Hitchcopse Pit, Oxfordshire, UK (latitude 51.69, longitude –1.35) on 2023-04-09 by netting. The specimen was collected and identified by Steven Falk (independent researcher) and preserved on dry ice.

The initial identification was verified by an additional DNA barcoding process according to the framework developed by Twyford *et al.* (2024). A small sample was dissected from the specimens and stored in ethanol, while the remaining parts of the specimen were shipped on dry ice to the Wellcome Sanger Institute (WSI). The tissue was lysed, the COI marker region was amplified by PCR, and amplicons were sequenced and compared to the BOLD database, confirming the species identification ( Crowley *et al.*, 2023). Following whole genome sequence generation, the relevant DNA barcode region is also used alongside the initial barcoding data for sample tracking at the WSI ( Twyford *et al.*, 2024). The standard operating procedures for Darwin Tree of Life barcoding have been deposited on protocols.io ( Beasley *et al.*, 2023).

### Nucleic acid extraction

The workflow for high molecular weight (HMW) DNA extraction at the WSI Tree of Life Core Laboratory includes a sequence of procedures: sample preparation and homogenisation, DNA extraction, fragmentation and purification. Detailed protocols are available on protocols.io ( Denton *et al.*, 2023b). The idCheGros1 sample was weighed and dissected on dry ice ( Jay *et al.*, 2023), and tissue from the abdomen was homogenised using a PowerMasher II tissue disruptor ( Denton *et al.*, 2023a).

HMW DNA was extracted in the WSI Scientific Operations core using the Automated MagAttract v2 protocol ( Oatley *et al.*, 2023). The DNA was sheared into an average fragment size of 12–20 kb in a Megaruptor 3 system ( Bates *et al.*, 2023). Sheared DNA was purified by solid-phase reversible immobilisation, using AMPure PB beads to eliminate shorter fragments and concentrate the DNA ( Strickland *et al.*, 2023). The concentration of the sheared and purified DNA was assessed using a Nanodrop spectrophotometer and Qubit Fluorometer using the Qubit dsDNA High Sensitivity Assay kit. Fragment size distribution was evaluated by running the sample on the FemtoPulse system.

RNA was extracted from abdomen tissue of idCheGros2 in the Tree of Life Laboratory at the WSI using the RNA Extraction: Automated MagMax™ *mir*Vana protocol ( do Amaral *et al.*, 2023). The RNA concentration was assessed using a Nanodrop spectrophotometer and a Qubit Fluorometer using the Qubit RNA Broad-Range Assay kit. Analysis of the integrity of the RNA was done using the Agilent RNA 6000 Pico Kit and Eukaryotic Total RNA assay.

### Hi-C preparation

Hi-C data were generated from the head and thorax tissue of idCheGros1 using the Arima-HiC v2 kit. In brief, frozen tissue (stored at –80°C) was fixed, and the DNA crosslinked using a TC buffer with 22% formaldehyde. After crosslinking the tissue was homogenised using the Diagnocine Power Masher-II and BioMasher-II tubes and pestles. Following the kit manufacturer's instructions, crosslinked DNA was digested using a restriction enzyme master mix. The 5’-overhangs were then filled in and labelled with biotinylated nucleotides and proximally ligated. An overnight incubation was carried out for enzymes to digest remaining proteins and for crosslinks to reverse. A clean up was performed with SPRIselect beads prior to library preparation.

### Library preparation and sequencing

Library preparation and sequencing were performed at the WSI Scientific Operations core. Pacific Biosciences HiFi circular consensus DNA sequencing libraries were prepared using the PacBio Express Template Preparation Kit v2.0 (Pacific Biosciences, California, USA) as per the manufacturer’s instructions. The kit includes the reagents required for removal of single-strand overhangs, DNA damage repair, end repair/A-tailing, adapter ligation, and nuclease treatment. Library preparation also included a library purification step using 0.8X AMPure PB beads and a size selection step to remove templates < 5 kb using AMPure PB modified SPRI. Samples were sequenced using the Sequel IIe system (Pacific Biosciences, California, USA). The concentration of the library loaded onto the Sequel IIe was within the manufacturer's recommended loading concentration range of 40–100 pM. The SMRT link software, a PacBio web-based end-to-end workflow manager, was used to set-up and monitor the run, as well as perform primary and secondary analysis of the data upon completion.

Poly(A) RNA-Seq libraries were constructed using the NEB Ultra II RNA Library Prep kit and RNA sequencing was performed on the Illumina NovaSeq 6000 instrument.

For Hi-C library preparation, DNA was fragmented to a size of 400 to 600 bp using a Covaris E220 sonicator. The DNA was then enriched, barcoded, and amplified using the NEBNext Ultra II DNA Library Prep Kit following manufacturers’ instructions. The Hi-C sequencing was performed using paired-end sequencing with a read length of 150 bp on an Illumina NovaSeq 6000.

### Genome assembly, curation and evaluation

***Assembly***

The HiFi reads were first assembled using Hifiasm ( Cheng *et al.*, 2021) with the --primary option. Haplotypic duplications were identified and removed using purge_dups( Guan *et al.*, 2020). The Hi-C reads were mapped to the primary contigs using bwa-mem2 ( Vasimuddin *et al.*, 2019). The contigs were further scaffolded using the provided Hi-C data ( Rao *et al.*, 2014) in YaHS ( Zhou *et al.*, 2023) using the --break option. The scaffolded assemblies were evaluated using Gfastats ( Formenti *et al.*, 2022), BUSCO ( Manni *et al.*, 2021) and MERQURY.FK ( Rhie *et al.*, 2020).

The mitochondrial genome was assembled using MitoHiFi ( Uliano-Silva *et al.*, 2023), which runs MitoFinder ( Allio *et al.*, 2020) and uses these annotations to select the final mitochondrial contig and to ensure the general quality of the sequence.

***Assembly curation***

The assembly was decontaminated using the Assembly Screen for Cobionts and Contaminants (ASCC) pipeline (article in preparation). Flat files and maps used in curation were generated in TreeVal ( Pointon *et al.*, 2023). Manual curation was primarily conducted using PretextView ( Harry, 2022), with additional insights provided by JBrowse2 ( Diesh *et al.*, 2023) and HiGlass ( Kerpedjiev *et al.*, 2018). Scaffolds were visually inspected and corrected as described by Howe *et al.* (2021). Any identified contamination, missed joins, and mis-joins were corrected, and duplicate sequences were tagged and removed. The curation process is documented at https://gitlab.com/wtsi-grit/rapid-curation (article in preparation).

***Evaluation of the final assembly***

A Hi-C map for the final assembly was produced using bwa-mem2 ( Vasimuddin *et al.*, 2019) in the Cooler file format ( Abdennur & Mirny, 2020). To assess the assembly metrics, the *k*-mer completeness and QV consensus quality values were calculated in Merqury ( Rhie *et al.*, 2020). This work was done using the “sanger-tol/readmapping” ( Surana *et al.*, 2023a) and “sanger-tol/genomenote” ( Surana *et al.*, 2023b) pipelines. The genome evaluation pipelines were developed using nf-core tooling ( Ewels *et al.*, 2020) and MultiQC ( Ewels *et al.*, 2016), relying on the Conda package manager, the Bioconda initiative ( Grüning *et al.*, 2018), the Biocontainers infrastructure ( da Veiga Leprevost *et al.*, 2017), as well as the Docker ( Merkel, 2014) and Singularity ( Kurtzer *et al.*, 2017) containerisation solutions. The genome was also analysed within the BlobToolKit environment ( Challis *et al.*, 2020) and BUSCO scores ( Manni *et al.*, 2021) were calculated.

Table 4 contains a list of relevant software tool versions and sources.

**Table 4.** Software tools: versions and sources.

Software tool	Version	Source
BlobToolKit	4.2.1	https://github.com/blobtoolkit/blobtoolkit
BUSCO	5.3.2	https://gitlab.com/ezlab/busco
bwa-mem2	2.2.1	https://github.com/bwa-mem2/bwa-mem2
Cooler	0.8.11	https://github.com/open2c/cooler
Hifiasm	0.16.1	https://github.com/chhylp123/hifiasm
HiGlass	44086069ee7d4d3f6f3f0012569789ec1 38f42b84aa44357826c0b6753eb28de	https://github.com/higlass/higlass
Merqury.FK	d00d98157618f4e8d1a9190026b19b47 1055b22e	https://github.com/thegenemyers/MERQURY.FK
MitoHiFi	3	https://github.com/marcelauliano/MitoHiFi
PretextView	0.2	https://github.com/wtsi-hpag/PretextView
purge_dups	1.2.5	https://github.com/dfguan/purge_dups
sanger-tol/genomenote	v1.0	https://github.com/sanger-tol/genomenote
sanger-tol/readmapping	1.1.0	https://github.com/sanger-tol/readmapping/tree/1.1.0
YaHS	1.2a.2	https://github.com/c-zhou/yahs

### Genome annotation

The BRAKER2 pipeline ( Brůna *et al.*, 2021) was used in the default protein mode to generate annotation for the *Cheilosia grossa* assembly (GCA_963082955.1) in Ensembl Rapid Release at the EBI.

### Wellcome Sanger Institute – Legal and Governance

The materials that have contributed to this genome note have been supplied by a Darwin Tree of Life Partner. The submission of materials by a Darwin Tree of Life Partner is subject to the **‘Darwin Tree of Life Project Sampling Code of Practice’**, which can be found in full on the Darwin Tree of Life website here. By agreeing with and signing up to the Sampling Code of Practice, the Darwin Tree of Life Partner agrees they will meet the legal and ethical requirements and standards set out within this document in respect of all samples acquired for, and supplied to, the Darwin Tree of Life Project. 

Further, the Wellcome Sanger Institute employs a process whereby due diligence is carried out proportionate to the nature of the materials themselves, and the circumstances under which they have been/are to be collected and provided for use. The purpose of this is to address and mitigate any potential legal and/or ethical implications of receipt and use of the materials as part of the research project, and to ensure that in doing so we align with best practice wherever possible. The overarching areas of consideration are:

•   Ethical review of provenance and sourcing of the material

•   Legality of collection, transfer and use (national and international)

Each transfer of samples is further undertaken according to a Research Collaboration Agreement or Material Transfer Agreement entered into by the Darwin Tree of Life Partner, Genome Research Limited (operating as the Wellcome Sanger Institute), and in some circumstances other Darwin Tree of Life collaborators.

## Data Availability

European Nucleotide Archive: *Cheilosia grossa*. Accession number PRJEB63511; https://identifiers.org/ena.embl/PRJEB63511 ( Wellcome Sanger Institute, 2023). The genome sequence is released openly for reuse. The *Cheilosia grossa* genome sequencing initiative is part of the Darwin Tree of Life (DToL) project. All raw sequence data and the assembly have been deposited in INSDC databases. Raw data and assembly accession identifiers are reported in Table 1 and Table 2.

## Author information

Members of the Natural History Museum Genome Acquisition Lab are listed here: https://doi.org/10.5281/zenodo.12159242.

Members of the University of Oxford and Wytham Woods Genome Acquisition Lab are listed here: https://doi.org/10.5281/zenodo.12157525.

Members of the Darwin Tree of Life Barcoding collective are listed here: https://doi.org/10.5281/zenodo.12158331

Members of the Wellcome Sanger Institute Tree of Life Management, Samples and Laboratory team are listed here: https://doi.org/10.5281/zenodo.12162482.

Members of Wellcome Sanger Institute Scientific Operations: Sequencing Operations are listed here: https://doi.org/10.5281/zenodo.12165051.

Members of the Wellcome Sanger Institute Tree of Life Core Informatics team are listed here: https://doi.org/10.5281/zenodo.12160324.

Members of the Tree of Life Core Informatics collective are listed here: https://doi.org/10.5281/zenodo.12205391.

Members of the Darwin Tree of Life Consortium are listed here: https://doi.org/10.5281/zenodo.4783558.
