# Supplementary material for: The genome sequence of a hoverfly, Cheilosia grossa (Fallén, 1817)
Source: Wellcome Open Res. 2024 Oct 21;9:616. [Version 1] doi: 10.12688/wellcomeopenres.23189.1 (PMC11574337; doi:10.12688/wellcomeopenres.23189.1)
Supplement: Peer review history

# Reviewer report: Reviewer response for version 1

**DOI:** 10.21956/wellcomeopenres.25539.r108152

**Published:** 2024-11-18

**Authors:** Xue Qing

**Affiliations:** 1 Nanjing Agricultural University, Nanjing, China

The *Cheilosia grossa *is an widely distributed species in Northern and Central Europe. Mitchell et al. present the first high quality genome assembly as well as the mitochondrial genome for this species. The genome assembly, gene prediction and annotation is comprehensive and professional, The newly generated genome can be a valuable reference for hoverfly evolution and ecology research. The manuscript is well written, the bioinformatic pipeline are well described and reproducible. I only have one comment on the background part: authors emphasis ecology aspect of the species, but as a genome assembly paper, it important to give 1-2 sentence overview on the current status of available genomes of hoverfly.  

 Overall, this work is important for the community, and can be accepted for indexing.

Are sufficient details of methods and materials provided to allow replication by others?

Yes

Is the rationale for creating the dataset(s) clearly described?

Yes

Are the datasets clearly presented in a useable and accessible format?

Yes

Are the protocols appropriate and is the work technically sound?

Yes

Reviewer Expertise:

Evolution, Phylogeny, Genome and metagenome, Nematode

I confirm that I have read this submission and believe that I have an appropriate level of expertise to confirm that it is of an acceptable scientific standard.

# Reviewer report: Reviewer response for version 1

**DOI:** 10.21956/wellcomeopenres.25539.r108154

**Published:** 2024-11-14

**Authors:** Komal Kumar Bollepogu Raja

**Affiliations:** 1 Baylor College of Medicine, Houston, TX, USA

The article by Mitchell et al. on *Cheilosia grossa* presents a comprehensive overview of both the ecological and genomic aspects of this hoverfly species. The authors effectively highlight the ecological role of this species in the introduction, providing a good background of the importance to generate genome assembly of this species.

 The authors have presented the genome with a high degree of quality, featuring a substantial length of 362.4 Mb in 12 scaffolds, and a high coverage of 355-fold, which is impressive. The authors have presented the methodology in good detail behind the genome assembly, curation, and evaluation. These details will allow for reproducibility. The BUSCO completeness of 96.9% suggest that  the quality of the assembly is high. The identification of the X chromosome and the mitochondrial genome, provide a valuable resource for further studies on *C. grossa*. The annotation of 20,196 protein-coding genes is also mentioned in the manuscript. Including a section on how these genetic insights could inform our understanding of *C. grossa*'s behavior, ecology, or evolutionary history would add an important layer of context. Overall, the article is an important contribution to both the ecological understanding and genetic characterization of *C. grossa*, with the genome sequence offering a promising foundation for future research.

Are sufficient details of methods and materials provided to allow replication by others?

Yes

Is the rationale for creating the dataset(s) clearly described?

Yes

Are the datasets clearly presented in a useable and accessible format?

Yes

Are the protocols appropriate and is the work technically sound?

Yes

Reviewer Expertise:

Single cell omics, Genomics, Bioinformatics, Neurobiology

I confirm that I have read this submission and believe that I have an appropriate level of expertise to confirm that it is of an acceptable scientific standard.
